# Comparison of ACEs reporting between clinic and telephone-based screening

**DOI:** 10.1371/journal.pone.0356066

**Published:** 2026-08-11

**Authors:** Rebecca Dudovitz, Hayden Ngan, Caryssa Lim Chang, Lorena Porras-Javier, Sitaram Vangala, Lizbeth Correa Mendoza, Janette Ortiz, Kary M. Calderon, Lindsey R. Thompson, Daniel Molina, Irene Aceves, Paul J. Chung

**Affiliations:** 1 Department of Pediatrics, Division of General Pediatrics, David Geffen School of Medicine at UCLA, Los Angeles, California, United States of America; 2 Kaiser Permanente Bernard J. Tyson School of Medicine, Pasadena, California, United States of America; 3 Department of Family Medicine, Keck School of Medicine of USC, Los Angeles, California, United States of America; 4 Division of General Internal Medicine- Health Services Research, David Geffen School of Medicine at UCLA, Los Angeles, California, United States of America; 5 211 Los Angeles, Los Angeles, California, United States of America; University of Ibadan, NIGERIA

## Abstract

**Background:**

Whether screening for Adverse Childhood Experiences (ACEs) can improve health outcomes may depend in part on screening being acceptable and accurate. Screening modality and context may be important determinants of these characteristics.

**Objective:**

We compared telephone ACE screenings by 211LA (a health and human services information and referral center) to self-completed screenings during well-child visits and identified family factors associated with differences in ACEs reporting.

**Participants and Setting:**

This is a secondary analysis of data from the intervention arm (n = 51) of a pilot randomized trial of telephone ACE care coordination via 211LA versus usual care at three Federally Qualified Health Centers. Children who had ≥ 1 ACE on the Pediatric ACEs and Related Life-events Screener (PEARLS) administered during a well-child visit and were randomized to the intervention were connected to 211LA 1–2 weeks after their clinic visit. 211LA then administered a subsequent telephone PEARLS screening.

**Methods:**

A within-subjects design examined whether scores differed between clinic and 211LA screening. Linear regressions tested whether trust in healthcare providers, parent stress, financial security, education, income, child age/sex, Latine ethnicity, and English proficiency predicted higher 211LA than clinic scores.

**Results:**

In paired t-tests, mean ACE scores were higher (2.08 vs. 1.35, p = 0.002) through 211LA than through clinics. In linear regressions, greater trust in healthcare providers (β = 0.64, p = 0.049), more parental stress (β = 0.06, p = 0.028), and speaking English “Well” versus “Very well” (β = 1.85, p = 0.043) were associated with a greater difference in 211LA versus clinic scores.

**Conclusions:**

Telephone-based screening may be effective for sensitive questions. Specific family factors may affect which screening approaches are preferable.

## Introduction

Adverse childhood experiences (ACEs) are potentially traumatic experiences during childhood (e.g., abuse, neglect, or household dysfunction) that may have significant impacts on health and development lasting well into adulthood [1]. Toxic stress affects attention and memory development, impacting future education, job opportunities, and income [2,3]. ACEs are associated with increased incidence of depression, diabetes, cancer, and heart disease [4,5]. ACEs also increase likelihood of high-risk health behaviors such as alcohol use, drug use, and unprotected sex [4–6]. These wide-ranging impacts create a disease burden some have estimated at $16.6 million in lifetime cost per affected individual and $428 billion annually in the United States [7].

Although ACEs present a significant issue for population health, studies show that ACEs may be preventable and their impacts on health mitigated [8–14]. ACEs may be prevented by providing resources such as family economic supports, life skills education, and youth mentorship programs [12,13]. Health impacts may also be mitigated if early identification of ACEs leads to healthcare services such as primary care and psychotherapy [12,14]. Although evidence remains mixed, some have called for universal ACE screening to identify affected families and connect them to services that may support their health [15].

Various approaches to ACE screening have been implemented. In many cases, ACE screening involves administering a written or oral questionnaire during a clinic visit, followed by a discussion of results and next steps. Previous epidemiological research found higher ACE reporting in telephone interviews compared to self-completed web/mail-based survey [16]. Additionally, aggregate clinical screening to determine an overall ACE score resulted in greater reporting than screening for each individual ACE item [17]. However, little else is known about how screening modality influences ACE reporting. Results are mixed for other types of sensitive screenings. Studies on screening for intimate partner violence (IPV) show no clear consensus on modality (verbal vs. written) or clinician (midwife vs. physician) [18,19]. One study on screening for depression with the Patient Health Questionnaire-2 (PHQ-2) found more positive screens with self-completed paper screening compared to verbal screening [20]; another study of the PHQ2 found that paper screening is comparable to smartphone versions [21].

Additional strategies for ACE screening are being studied to alleviate the burden on clinics, especially those serving communities facing structural disadvantages and that are disproportionately impacted by ACEs. One method under investigation is the use of telephone-based screening, service navigation, and case management via 211LA, a non-profit health and human services organization that is part of the national 2-1-1 call center network and maintains a database of over 50,000 health services in Los Angeles (LA) County. Our team conducted a small pilot randomized controlled trial in pediatric clinics already performing in-person clinic screening to 1) evaluate the feasibility and acceptability of telephone-based ACE screening and 2) compare receipt of ACEs-related services through usual care plus 211LA care coordination vs. usual care alone. [22]

This manuscript takes advantage of the fact that families in the intervention group of the trial completed ACEs screening both in-person during their clinic visit and by telephone during a subsequent call with 211LA. We examine whether families reported higher or lower ACE scores using 211LA telephone-based screening versus in-person clinic screening, and whether family characteristics are associated with differences in reported ACE scores between 211LA and clinic screening. Given the mixed results on modality for other sensitive screenings, we hypothesized that participants would be more likely to report ACEs on a typical self-completed paper screening in clinic than during a telephone-based 211LA screening, theorizing that the close caregiver-provider relationship would make parents more willing to report to clinicians than to 211LA care coordinators.

## Materials and methods

This study was reviewed and approved by the UCLA IRB (IRB#22–000405). The UCLA IRB waived the requirement for signed informed consent/parental permission for the research under 45 CFR 46.117. All study activities were completed by phone. Participants were sent a study information sheet. Oral consent was obtained for all participants, witnessed by a research associate, and documented in the study REDCap database, as approved by our IRB.

### Study design

Study data were drawn from the intervention arm of a randomized trial of telephone-based ACEs care coordination delivered by 211LA (clinicaltrials.gov NCT number 05567250). For the parent trial, we recruited 102 of 158 eligible families (64.6%) of children aged 0–11 with at least 1 ACE on Part 1 of the Pediatric ACEs and Related Life-events Screener (PEARLS) administered during well-child visits at three participating Federally Qualified Health Centers (FQHCs) in Los Angeles, California between October 5, 2022 and August 7, 2023. Part 1 of the PEARLS tool contains the original 10 ACE questions related to past traumatic experiences that are known to impact long term health and wellbeing, and Part 2 of the PEARLS contains questions about current social factors that may impact current health status. [23,24] After completing a baseline survey, families were randomized 1:1 to the intervention versus control group at the individual patient level, stratified by clinic site. Only families randomized to the intervention (n = 51) received a subsequent telephone-based PEARLS screening by 211LA 1–2 weeks later, followed by referrals to community-based ACEs-related service providers and subsequent care coordination. This sub-analysis examines these 51 families in the intervention group.

### Participants

Parents or caregivers of children aged 0–11 who screened positive for at least one ACE on PEARLS Part 1 during a clinic visit at one of 3 FQHCs were enrolled in the study. Other eligibility criteria included the parent being at least 18 years of age and able to complete a telephone survey in English or Spanish. Families in the child welfare system were excluded, as they already receive additional evaluation and referral for ACEs, as were families with siblings already enrolled in the study.

For eligible participants, in accordance with our study protocol approved by the UCLA Institutional Review Board, an oral consent script was read, and verbal consent was obtained by a study staff member via telephone. The study information sheet was texted or emailed to participants, based on participant preference. After obtaining informed consent, a baseline survey was administered via telephone. Parents were then randomized and, if in the intervention arm, connected to 211LA care coordinators via a three-way phone call when possible; when not possible, their contact details were provided for 211LA to contact. Families then underwent telephone-based ACE screening by 211LA followed by referrals to ACEs-related services. All families continued to receive usual care at their primary care clinic, including any clinic-provided referrals to ACEs-related services.

### Quantitative measures

211LA and all participating clinics screened for ACEs using the PEARLS child tool for ages 0–11. In clinics, parents or caregivers were given the paper version of the de-identified PEARLS screener in English or Spanish during a well-child visit. In the de-identified format, although parents were asked about each ACE, they only needed to report the total number of positive ACEs (i.e., the ACE score) without indicating which specific ACE applied to their child. In 211LA screening, the care coordinator asked about each ACE included in PEARLS; parents then indicated if their child had experienced it. The total number of ACEs identified was communicated to the research team, but not the specific ACEs participants experienced. 211LA bilingual care coordinators conducted the screening in English or Spanish based on the parent’s preferred language.

The baseline survey included basic demographic information, along with several family outcomes including parental stress (Parent Stress Scale) [25], financial wellbeing (CFPB Financial Wellbeing Scale) [26], and caregiver-clinician relationship (Protective Factors Survey 2.0) [27]. The former two domains were selected because we hypothesized that increased distress (as indicated by high stress or poor financial wellbeing) could make parents more willing to disclose ACEs to 211LA to receive help. The last domain was selected because we hypothesized that greater trust in the caregiver-clinician relationship would make parents more willing to report ACEs in clinic versus through 211LA.

### Qualitative interviews

We conducted post-hoc qualitative interviews to understand parent experiences with ACE screening. A subset of participants was selected for interviews and recruited via phone. Participants were purposively selected to highlight differences in ACE score between clinic and 211LA screening and to ensure roughly even distribution across clinic sites, self-rated usefulness of the ACE screening, and ACE risk level. Self-rated usefulness of ACE screening was based on response to the question, “Please rate how useful the [ACE] screening was where 1 is ‘Not at all useful’ and 10 is ‘Extremely useful’.” ACE risk level was assessed based on the PEARLS Part 1 ACE score and the presence of associated health conditions, according to the ACEs Aware Toxic Stress Risk Assessment Algorithm [24]. An ACE score of 0 (excluded from our study) is categorized as low risk; 1–3 without associated health conditions is intermediate risk; and 1–3 with associated health conditions or 4+ with or without associated health conditions is high risk. During the interview, parents were asked to reflect on why some participants might have reported different ACE scores when screened in clinic versus over the phone.

### Quantitative analysis

Within-subject differences in ACE scores between 211LA and clinic-based screening were evaluated using a two-sided paired t-test. Although we hypothesized that clinic ACE scores would be higher than 211LA ACE scores, we chose a 2-sided test to more conservatively evaluate any difference between scores. Because the intervention group in this pilot sample was small, multivariable regressions were infeasible. Unadjusted linear regressions were used to test whether trust in healthcare providers, parent stress, financial security, education, income, child age/sex, Latine ethnicity, and English proficiency were associated with a greater difference in 211LA scores relative to clinic ACE scores (i.e., 211LA score minus clinic score). A significance level of 0.05 was used. Statistical analyses were conducted in R v. 4.1.0 (http://www.r-project.org/).

### Qualitative analysis

Spanish interviews were analyzed in Spanish, and several illustrative quotes were translated for publication. A codebook was created to identify overarching themes in parent experiences of ACE screening and perceived differences between clinic and 211LA screening. We used thematic analysis to identify and define general themes from the codes, then consolidated those themes into summaries for further discussion. Qualitative analysis was conducted using Dedoose. [28] Coding was conducted by three coders in Dedoose. To ensure inter-rater reliability and agreement between coders, coders were required to score a minimum kappa of 0.60 for a random sample of codes. The kappa scores of the coders ranged from 0.80 to 0.89.

## Results and discussion

### Results

#### Study population.

All 51 intervention arm families were included in the analysis (Table 1). Mean child age was 46.5 months; 78.4% of families identified as Latine; 74.5% of parents reported speaking English “Very well.” Over 70% of families reported yearly income less than $40,000 in 2021; 98% of families were insured through Medicaid, with a small percentage being uninsured.

**Table 1:** Characteristics of sampled children and parents (n = 51).

Characteristic	No. (%) or mean (SD)
Child age in months	46.5 (35.5)
Child sex	
Male	54.9%
Female	45.1%
Latine ethnicity	78.4%
Health insurance	
Medi-Cal	98.0%
None	2.0%
At least one parent born outside US	41.2%
At least one parent completed high school	45.1%
Income	
< $20,000	32.0%
$20,000-$39,999	40.0%
$40,000-$69,999	18.0%
> $70,000	6.0%
English proficiency	
Very well	74.5%
Well	5.9%
Not well	11.8%
Not at all	7.8%
Protective Factors Survey 2.0: Caregiver Practitioner Relationship	5.1 (2.1)
Parent Stress Scale Score (18–90)	33.0 (7.5)
CFPB Financial Wellbeing Scale (0–100)	52.7 (13.2)

#### Mean ACE scores.

**Fig 1:**
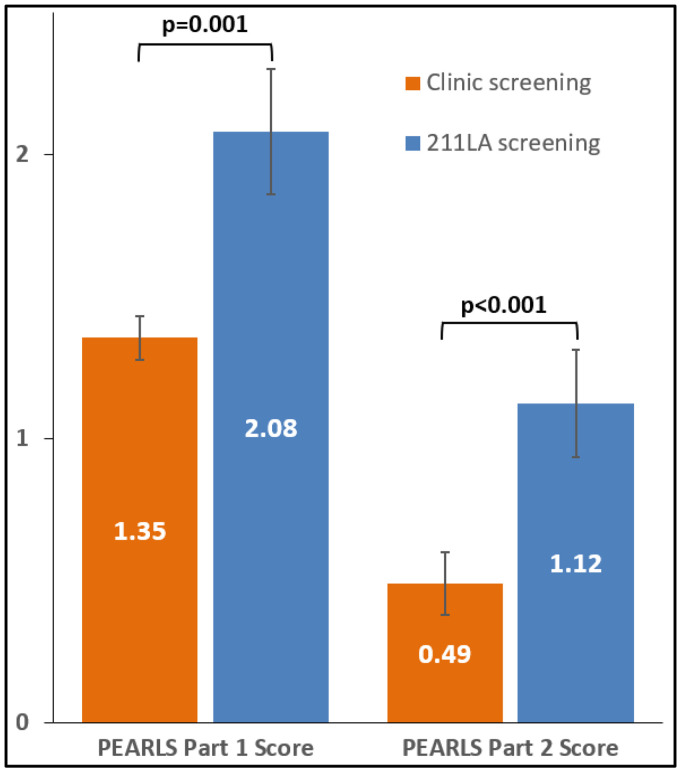
Paired t-test testing agreement in PEARLS scores within individuals between clinic and 211LA. Mean PEARLS Part I and Part 2 scores are shown for the clinic administered ACEs screening in orange and the 211LA ACEs screening in blue with 95% confidence intervals. P-values result from paired t-tests testing whether scores differed within individuals across these settings.

The mean ACE score from PEARLS Part 1 was 1.35 (95% CI 1.20–1.51) in clinic screening and 2.08 (95% CI 1.64–2.51) with 211LA screening; the mean score from PEARLS Part 2 was 0.49 (95% CI 0.27–0.71) in clinic screening and 1.12 (95% CI 0.75–1.48) with 211LA screening (Fig 1). In a paired t-test, PEARLS PART 1 ACE scores in 211LA screening were significantly higher than in clinic screening (difference = 0.73, 95% CI: 0.29–1.16, p = 0.002).

#### Qualitative interviews.

Thematic analysis of parents’ answers on perceived differences in ACE screening between 211LA and clinic revealed several key themes. Parents attributed the difference in ACE reporting to greater convenience, anonymity, privacy, focus, and the prospect of getting help from 211LA (Table 2).

**Table 2:** Themes and illustrative quotes of perceived differences between 211LA and clinic screening.

Theme	Illustrative quotes
Greater convenience in phone screening	“Over the phone is convenient just because I’m a full-time mom and a full-time employee and I’m juggling so much.”
	“I can be on the go or I could be on my break. I don’t have to literally drive to the clinic and go talk to them… I mean, I go to the clinic for my little boy, but I don’t really go to his clinic outside of him being sick, you know?”
Greater anonymity in phone screening	“If you’re in person at the clinic, you might not say everything that you want to say. Over the phone you’re talking to someone you don’t know. You don’t see them, so maybe so you become a little bit more vulnerable and open.”
	“Over the phone it is a little bit more comfortable… mainly because you don’t see how nervous I am. You don’t see me fidgeting with my hands.”
Greater privacy in phone screening	“I think [parents report fewer ACEs in clinic] because [of] the level of comfort... [They’re] not comfortable answering this question here and now, and they decide either to open up or shut down.”
Greater focus in phone screening	“In that appointment, you gotta fill this paper out while talking to the doctor, taking care of your kid, … doing this, doing that. So maybe we we’re not properly reading the questions right, [compared to over the phone] where we can actually take the time to think about what we’re saying and properly answer the questions.”
	“[There are] not many places [to] sit and… take the time to think about your answers… because the appointment’s [only] 10 minutes [long], if that.”
Prospect of getting help from 211LA	“I was a little bit more embracing of [211LA] because I [thought], ‘OK, this is something that’s [here] to help.’”
	“I feel like the clinic is just so much authority [while 211LA is] more open, more helpful… I know they’re both supposed to be helpful, but when you go to an organization [211LA], it’s much more like a person knows me. I know I need that resource, so I’m going to be truthfully honest with them [211LA], because they’re there to give me that [resource]. [But at] the clinic it’s like, ‘What can you do for me? Why am I going to tell you my struggles if that’s where it’s going to end?’”

Parents felt screening in clinic was less comfortable for several reasons: needing to provide sensitive information in a non-private setting, being distracted by their children, and needing to answer other questionnaires given by the clinic. Meanwhile, screening over the phone afforded the convenience of flexibility, as parents could postpone screening and ask to be called back later. It also allowed greater anonymity and privacy, as parents could take the call from a private location and discuss sensitive issues without seeing someone face-to-face. Completing it over the phone also allowed better focus, as they didn’t have to simultaneously keep their children entertained in the clinic. Parents also felt more likely to receive help through referrals from 211LA. Some parents felt “more embracing of [211LA]” because they saw 211LA as a “follow-up,” going “hand-in-hand” with the clinic visit.

Although most parents found screening tolerable, helpful, and worthwhile regardless of modality, several parents cited fear of being reported to Child Protective Services or having their children taken away as reasons not to report to either 211LA or the clinic.

#### Factors associated with a greater difference in 211LA ACE scores versus clinic scores.

In linear regressions, greater trust in providers (β = 0.64, 95% CI: 0.01–1.27, p = 0.049), more parental stress (β = 0.06, 95% CI: 0.00–0.12, p = 0.028), and speaking English “Well” versus “Very well” (β = 1.85, 95% CI: 0.11–3.59, p = 0.043) were associated with higher 211LA scores relative to clinic PEARLS Part I ACE scores (Table 3). Other factors that were tested but showed no significant relationship included financial wellbeing, income in 2021, at least one parent born outside the US, at least one parent with a high school education or greater, Latine ethnicity, child age in months, and child gender.

**Table 3:** Unadjusted association of family contextual factors with difference between PEARLS Part I ACE scores via 211LA versus clinic screening (n = 51)*.

Predictor variable (Reference group)	Regression coefficient (SE)	P
**Trust in providers (0–12)** *Protective Factors Survey 2.0: Caregiver Practitioner Relationship*	0.64 (0.32)	**0.049**
**Parent Stress Scale Score (18–90)**	0.06 (0.03)	**0.028**
**CFPB Financial Wellbeing Scale (0–100)**	−0.01 (0.02)	0.608
**Spoken English Proficiency** (Ref = Very Well)		
Well	1.85 (0.89)	**0.043**
Not Well	−1.15 (0.65)	0.085
Not at All	−0.82 (0.78)	0.301
**Income in 2021** (Ref = Less than $20,000)		
$20,000 to $39,999	−0.11 (0.52)	0.831
$40,000 to $69,999	−0.37 (0.65)	0.574
$70,000 or Greater	0.19 (0.98)	0.849
N/A	−1.31 (1.17)	0.268
**At Least One Parent Born Outside US**	0.26 (0.45)	0.564
**At Least One Parent with Greater than High School Education**	1.03 (0.74)	0.169
**Latine Ethnicity**	−0.47 (0.54)	0.391
**Child Age in Months**	0.00 (0.01)	0.467
**Child Gender** (Ref = Female)	−0.85 (0.43)	0.055

*Outcome is defined as 211LA ACEs score minus clinic ACE score.

### Discussion

The study found a significant positive difference between PEARLS ACE scores from 211LA and clinic screening. Parents attributed the difference in reporting to greater convenience, anonymity, privacy, focus, and prospects for getting help. Unadjusted linear regressions showed that greater trust in providers, more parental stress, and speaking English “Well” versus “Very well” were associated with a larger positive difference between 211LA and clinic ACE scores.

#### Parents reported more ACEs to 211LA than to clinics.

The a priori hypothesis for our primary aim was that parents would be more likely to report ACEs (Part 1 of the PEARLS) and social determinants of health (Part 2 of the PEARLS) to their clinicians than to 211LA care coordinators, based on assumed trust between parent and provider; thus, the finding that ACE scores were significantly higher in 211LA screening relative to clinic screening was somewhat surprising. This result suggests that screening context may influence ACE reporting or that some screening modalities may result in more positive screens. With respect to modality, a study of population-based surveys suggested that telephone ACE screening may increase ACE reporting compared to self-completed web-based screening, which the authors attributed to trust developed through verbal communication [12]. Another study, however, showed more positive PHQ-2 screens with self-completed paper screening compared to verbal screening [20]. Most studies on screening of sensitive topics show no clear difference between modalities [18,19,21].

The finding that phone screening results in higher ACE scores compared to self-completed paper surveys in clinics is novel to the literature. Existing literature suggests that de-identified in-person ACE screening leads to more positive responses on both Part 1 and Part 2 of the PEARLs screener than identified in-person ACE screening [17], but prior studies have not examined how phone screening affects reporting. Evaluations of screening of sensitive topics over the phone are scant: multiple studies have found smartphone apps effective at screening for depression, but no studies exist on verbal depression screening over the phone [29–31]. Multiple studies have also examined telemedicine interventions for patients with known depression or intimate partner violence (IPV), but not telemedicine screening [32–34].

There were many differences between 211LA and clinic screening, including modality (telephone vs. in-person, verbal vs. written, administered vs. self-completed, identified vs. de-identified), location (at-home vs. in-clinic), agenda (screening for and responding only to ACEs vs. addressing multiple clinical issues), and timing (211LA screening happened after clinic screening). It is unclear which aspects of 211LA screening contributed to the difference in reporting, but parents reported clear differences between 211LA and clinic screening in qualitative interviews.

Better convenience, anonymity, privacy, and focus are all reasonable explanations for a difference in reporting between 211LA and clinic screening. However, these factors may be more inherent to phone screening generally than to 211LA. Thus, it is plausible that clinics could offer phone screenings to increase access to ACE screening for underserved patients without 211LA involvement. On the other hand, using phone screening without involving 211LA would not provide the final parent-endorsed advantage of 211LA care navigation for referrals and follow-up, which in initial analyses resulted in greater receipt of services about four months after baseline. [22]

It is not immediately clear why some parents felt the prospect of getting help motivated them to report more through 211LA than in clinic, given that both 211LA and FQHCs can refer families to appropriate services. One possible explanation is that the purpose of screening was clearer with 211LA than with the clinic. Ideally, any ACE screening starts by explaining that a core purpose of screening is to provide help, including via referral to services. It is unclear how often this was explained during busy FQHC visits with multiple objectives. In contrast, the process of enrollment and warm hand-off from the study team to 211LA care coordinators was standardized and presented to parents as focused on getting appropriate help.

#### Factors associated with larger differences between 211LA and clinic ACE scores.

Higher parent stress was associated with greater reporting to 211LA. Parent stress may reflect a greater need for supportive interventions, perhaps making them more willing to report ACEs in general. However, it is unclear why greater parent stress might motivate parents to report specifically to 211LA over their clinic. Speculatively, perhaps the potentiating effect of higher parent stress on reporting to 211LA is mediated by the convenience, anonymity, and other benefits of 211LA screening. Surprisingly, other factors that we considered proxies for “level of need” such as income and financial wellbeing were not associated with higher reporting to 211LA. That parent stress was associated with reporting while income was not might indicate that a family’s perceived need for services is less dependent on available resources than on parents’ ability to cope with stress related to ACEs.

It is also unclear why parents with greater trust in their healthcare providers would report more ACEs to 211LA than in clinic. We had theorized that trust in providers would increase reporting in clinics, rather than to 211LA. Of note, clinic screening was generally done via self-completed survey and not necessarily face-to-face with a clinician, so rapport and trust may have played less of a role. Speculatively, one possibility is that parents saw 211LA as being part of the clinic follow-up, so their trust toward providers was also transferred to 211LA care coordinators. Several parents noted that 211LA felt like a follow-up to the in-clinic survey. Thus, parents with greater trust in their clinicians may have reported more during “follow-up” with 211LA. The effect size was large enough to be clinically significant – a one-unit increase of a parent’s trust in providers on any one of three questions was associated with a 0.64-point difference in reported ACE score between 211LA and clinic. For example, a parent who answered “Strongly Agree” instead of “Agree” to a statement like “I feel like staff here understand me” could report one additional ACE, which might alter the clinical response and eligibility for other services.

English proficiency was not associated with any difference in ACE reporting except for between speaking English “Very well” and “Well”. One explanation is that 211LA offered Spanish-speaking coordinators to all patients who spoke it, while in-clinic interpretation may often be reserved only for patients who speak little English, leaving patients who speak English “well” but not “very well” in a language doughnut hole. Communicating in one’s preferred language may be particularly important when discussing sensitive topics such as ACEs.

Together, these associations suggest that screening modality and context might be important for ACEs identification, and that the “best” screening approach may vary by patient characteristics.

One major limitation of this study was that all participants received 211LA screening just two weeks after clinic screening and that only those reporting some ACE exposure during the clinic screening participated in this study. Indeed, referral to 211LA was intended to support mitigation of toxic stress that is known to occur in the setting of a positive ACE screen and is thought to be essential for potentially achieving clinical benefit from ACE screening. Reporting could have been higher on the second screening due to greater recall when prompted again or greater familiarity or comfort with the questionnaire items. The latter seems especially plausible, as invasive questions on sensitive issues may be more palatable after repeated exposure. This is in line with a study on postpartum depression that found an increase in positive screens when repeating screening 6 months later, although this may be attributed to the natural progression of postpartum depression itself [35].

Another limitation is the small sample size of this pilot (n = 51). We were unable to perform multivariable analysis, making the results subject to confounders. Thus, it is best to consider these results hypothesis-generating only rather than confirmatory, warranting further study of the effect of telephone screening and screening context on ACE reporting. Finally, participants were drawn from 3 Federally Qualified Health Centers in Los Angeles. Although the demographic characteristics are consistent with low-income families impacted by ACEs in Southern California, results may not generalize to other populations.

#### Significance.

Our main exploratory finding suggests that screening for ACEs over the phone outside of a clinic visit may be effective at identifying ACE exposure—with the ultimate goal of prompting an effective response to a positive screening result. If confirmed in larger trials, this finding opens potential opportunities to build and test systems for ACE identification and management that only minimally burden busy primary care clinics. Telephone screening and response activities may require fewer resources and personnel and be easier to scale up than a clinic-based screening program.

Such systems may also have the advantage of reaching more vulnerable patient populations, which may be less likely to present for preventative healthcare [3,36] Telephone screening may be especially helpful for families with difficulty accessing care due to cost, insurance, scheduling, or transportation issues. This is an important consideration for those who continue to investigate optimal strategies for prevention of, identification of, and responses to ACEs and toxic stress.

## Conclusions

We found that parents reported more ACEs via phone screening by 211LA than in clinic via self-completed surveys, which may be due in part to greater convenience, anonymity, privacy, focus, and the prospect of getting help from 211LA. Greater trust in providers, more parental stress, and speaking English “Well” versus “Very well” were associated with higher 211LA scores relative to clinic ACE scores. Telephone-based screening may be effective for sensitive questions, and several family or patient-clinic relationship factors may affect which screening approaches are preferable. Systems like 211LA may be a viable adjunct to clinic screening for and responding to ACEs and toxic stress, enabling broader acceptance, uptake, and impact among children and families.
